# Calcineurin B homologous protein 3 binds with high affinity to the CHP binding domain of the human sodium/proton exchanger NHE1

**DOI:** 10.1038/s41598-018-33096-5

**Published:** 2018-10-04

**Authors:** Simon Fuchs, Sierra C. Hansen, Marie Markones, Evgeny V. Mymrikov, Heiko Heerklotz, Carola Hunte

**Affiliations:** 1 Institute for Biochemistry and Molecular Biology, ZBMZ, Faculty of Medicine, University of Freiburg, D-79104 Freiburg, Germany; 2 Faculty of Biology, University of Freiburg, D-79104 Freiburg, Germany; 3 Department of Pharmaceutical Technology and Biopharmacy, University of Freiburg, D-79104 Freiburg, Germany; 4 BIOSS Centre for Biological Signalling Studies, University of Freiburg, D-79104 Freiburg, Germany; 5 Leslie Dan Faculty of Pharmacy, University of Toronto, Toronto, Canada

## Abstract

The Na^+^/H^+^ exchanger NHE1 is critical for cell vitality as it controls intracellular pH and cell volume. Its functionality is influenced by calcineurin B homologous proteins (CHPs). The human isoform CHP3 is important for transport of NHE1 to the plasma membrane and for its activity. Here, we characterized the binding interaction of human CHP3 with the regulatory domain of NHE1. The exact binding site of CHP3 was previously debated. CHP3 as well as both regions of NHE1 in question were produced and purified. CHP3 specifically formed stable complexes with the CHP-binding region (CBD) of NHE1 (residues 503–545) in size-exclusion chromatography (SEC), but not with the C-terminal region (CTD, residues 633–815). CTD was functional as shown by Ca^2+^-dependent binding of calmodulin in SEC analysis. CHP3 bound with high affinity to CBD with an equilibrium dissociation constant (*K*_D_) of 56 nM determined by microscale thermophoresis. The high affinity was substantiated by isothermal calorimetry analysis (*K*_D_ = 3 nM), which also revealed that the interaction with CBD is strongly exothermic (ΔG° = −48.6 kJ/mol, ΔH = −75.3 kJ/mol, −TΔS° = 26.7 kJ/mol). The data provide insights in the molecular mechanisms that underlie the regulatory interaction of CHP3 and NHE1 and more general of calcineurin homologous proteins with their target proteins.

## Introduction

A constant intracellular pH is critical for cell vitality and is thus tightly controlled in living cells^1^. Important for this fundamental cellular process is the interaction of the sodium/proton exchanger NHE1 with calcineurin B homologous proteins (CHPs) of which three isoforms exist.

NHE1 is embedded in the cytoplasmic membrane and transports excess protons in exchange to sodium ions into the extracellular space^2–4^. It is a secondary active transport which is driven by the inward sodium ion gradient generated by Na^+^/K^+^ ATPase activity. NHE1 is ubiquitous in human cells. The mature protein consists of an N-terminal transmembrane domain that facilitates the ion exchange and a cytoplasmic C-terminal domain which acts as a hub for regulatory protein-protein interactions and posttranslational modifications^5,6^. The astonishing regulatory network reflects the central role of NHE1 to maintain cell vitality upon changes in the metabolic and developmental cell state and in the cellular environment. The integrative control of NHE1 activity is essential for cell proliferation and differentiation. NHE1 malfunction is implicated in a variety of pathophysiological conditions. Enhanced NHE1 activity for instance triggers cell death during episodes of ischemia-reperfusion in cardiac and neural tissues and has been linked to cardiac hypertrophy and heart failure^7^. Inherited loss-of-function mutations of NHE1 result in the Lichtenstein-Knorr syndrome, a recessive neurologic disorder characterized by cerebellar ataxia and severe sensorineural hearing loss^8^.

The calcineurin B homologous proteins CHP1, CHP2, and CHP3 are important both for trafficking of NHE1 to the plasma membrane and for influencing NHE1 half-life and transport activity^9^. They belong to the large class of calcium-ion binding EF-hand proteins. The functional importance of their interaction with NHE1 is stressed by the recent finding that syndromic ataxia-deafness is not only linked to loss-of-function mutations of NHE1, but also to mutations of CHP1 that impair NHE1 membrane targeting^10^.

CHP3 was first identified in testis as well as brain tissue of rats, and then termed tescalcin^11,12^. It promotes the biosynthetic maturation, cell surface stability and transport activity of NHE1^13^. It has additional binding partners including subunit 4 of the COP9 signalosome (CSN4) and the protein kinase glycogen-synthase kinase 3 (GSK3)^14,15^. The nature of the interaction between CHP3 and NHE1 is debated^16^. It was initially suggested that CHP3 binds to the C-terminal region of NHE1’s regulatory domain, as addition of CHP3 decreased the fluorescence of a cascade-blue labelled C-terminal NHE1 fragment comprising residues 633–815^17^. In contrast, CHP3 interacted in GST-mediated pull-down experiments with the N-terminal region of the regulatory domain (residues 503–545) but not with the C-terminal region (residues 633–815). GST-fusion proteins of the respective NHE1 fragments were used for this study^18^.

In addition to this conflicting information, there are no data available regarding the energetics of the interaction. The latter is also the case for the interaction between NHE1 and CHP1 or CHP2, though the characterization of these interactions is more advanced. Structures of CHP1 and CHP2 were determined by NMR and x-ray crystallography, respectively^19,20^. They revealed not only as expected two globular domains, the so-called N-lobe and C-lobe, but resolved also the interaction with the N-terminal region of the regulatory domain of NHE1. Both structures were obtained in complex with a peptide that included the CHP binding region (CBD). The peptide forms an amphipathic helix which binds to a hydrophobic groove between N- and C-lobe. The C-terminal region of NHE1 also features an amphipathic helix, which interacts with a non-canonical elongated form of calmodulin (CaM)^21^. A high affinity (*K*_D_ = 20 nM) and a low affinity (*K*_D_ = 350 nM) binding site for CaM were reported for the C-terminal region of the regulatory domain of NHE1 and the binding interaction is calcium ion dependent^22^.

Here, we demonstrate with purified proteins that CHP3 binds specifically to the N-terminal region of the regulatory domain of NHE1 (residues 503–545, termed CBD) and not to its C-terminal region (residues 633–815, termed CTD). Characterization of the energetics of the binding interaction by microscale thermophoresis and isothermal calorimetry demonstrated a high binding affinity with equilibrium dissociation constant (*K*_D_) in the nanomolar range. CHP3 binding is more enthalpy-driven. The data enhance our understanding of the molecular mechanisms that underlie the regulatory interaction of CHP3 and NHE1 and are of general interest for the interaction between calcineurin B homologous proteins and their target proteins.

## Results

### CHP3 binds to the CBD region of NHE1

**Figure 1:**
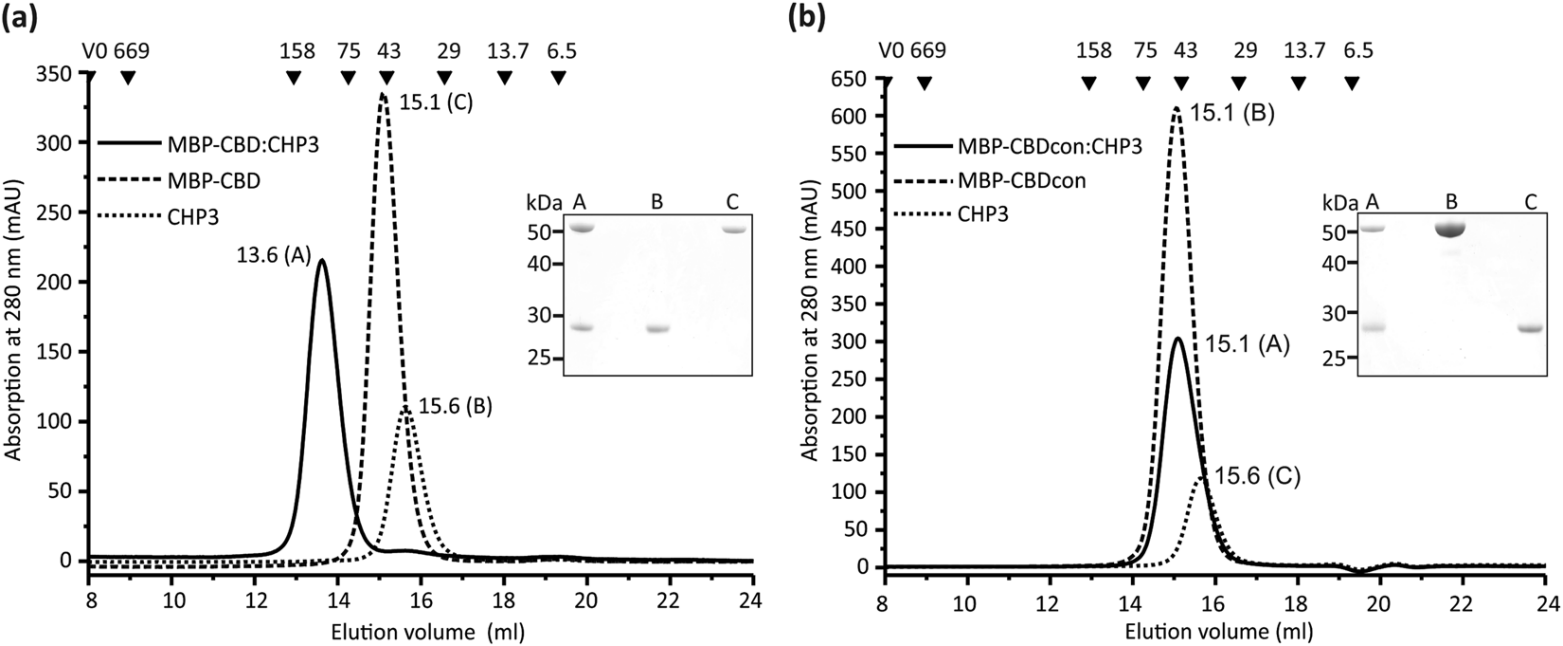
Interaction between CHP3 and the CBD region of NHE1 probed *in vitro* by size exclusion chromatography. (**a**) Purified CHP3 and MBP-CBD^NHE1^ were separated alone and as equimolar mixture using a Superdex 200 10/300 GL column. Shown are the three superimposed chromatograms. Numbers close to the elution peaks indicate the respective elution volume in ml. Black triangles with numbers above indicate positions of the elution peaks of standard proteins used for calibration and their respective molecular mass in kDa. V0 marks the void volume. SDS-PAGE analysis of the respective elution peaks (A-C) was performed and a section of the Coomassie-stained gel is shown as inset. Complete formation of stable complex of CHP3 and MBP-CBD is clearly demonstrated by a single elution peak with lower retention volume and the presence of both proteins in the elution fraction. (**b**) The control experiment was performed as in (**a**) with CHP3 and MBP-CBDcon. The elution profile of the equimolar mixture did not shift to lower retention volume indicative of lack of complex formation. Both proteins are present in the fractions of this elution peak, as CHP3 and MBP-CBDcon have similar retention volumes.

In order to clarify how CHP3 binds to the regulatory domain of human NHE1, *in vitro* binding studies with both regions in question, CBD (NHE1_503–545_) and CTD (NHE1_633–815_), and human CHP3 were conducted. The three proteins were produced by heterologous expression. Highly pure and homogenous samples of CHP3 comprising a carboxy-terminal hexa-histidine tag (abbreviated here CHP3) were prepared by immobilized-metal-affinity chromatography (IMAC) and consecutive size-exclusion chromatography (SEC) with an average yield of 40 mg/l cell culture (Fig. 1a). CBD was produced in fusion with maltose-binding protein at its amino-terminus (MBP-CBD). Purification by IMAC and consecutive SEC provided a pure and homogenous protein preparation as probed by analytical SEC and SDS-PAGE analysis (Fig. 1a). The typical yield was 30 mg purified MBP-CBD per liter of cell culture. For binding analysis, purified proteins were mixed in equimolar ratio and complex formation was probed using analytical SEC. Single symmetrical elution peaks for CHP3 and MBP-CBD were resolved with corresponding apparent molecular masses of 38 kDa and 49 kDa, respectively. Apparent mass and theoretical (48 kDa) molecular mass are in good agreement for MBP-CBD. CHP3 migrated slightly faster than expected from its theoretical molecular mass of 28 kDa. This may derive from a larger hydrodynamic radius of the protein, as structures of related calcineurin B homologous proteins, namely of CHP1^20,23^ and of CHP2^19^ showed an elongated bi-lobed shape. MBP-CBD and CHP3 formed a stable complex that was resolved as a single symmetrical elution peak. Both, CHP3 and MBP-CBD were present in the peak fractions as shown by subsequent SDS-PAGE analysis (Fig. 1a, inset). The corresponding apparent molecular mass of 95 kDa suggests a 1:1 complex and the lack of additional peaks supports a defined bi-molecular stoichiometry.

In order to exclude non-specific interactions between CHP3 and the MBP region of MBP-CBD, the experiment was also carried out with a MBP-CBD control protein (MBP-CBDcon), in which hydrophobic residues in the core region of CBD were substituted with polar residues by site-directed mutagenesis (F_526_LDHLL_531_ to Q_526_QDHQQ_531_). These substitutions were previously shown to disrupt the interaction between NHE1-peptides fused to GST and CHP3 in pull-down experiments from reticulocyte lysates^13^. The substitutions did neither affect production and purification of the protein (yield 30 mg protein/l cell culture) nor the elution profile in size-exclusion chromatography. The retention volumes of MBP-CBDcon and MBP-CBD were identical (Fig. 1a,b). Elution profiles of the stoichiometric mixture of MBP-CBDcon and CHP3 clearly showed the lack of complex formation. No larger complex was formed, but both proteins co-eluted due to their overlapping individual elution peaks. The control experiment supports the specific nature of the interaction between MBP-CBD and CHP3.

### The CTD region of NHE1 binds CaM and not CHP3

Next, we tested whether CHP3 also interacts with the CTD region of NHE1 (NHE1_633–815_). Hexa-histidine tagged CTD as well as the known CTD binder calmodulin (CaM) were produced and purified by IMAC followed by SEC. The final yield of pure protein was on average 12 mg and 6 mg protein/l cell culture for CTD and CaM, respectively. Binding analysis was performed by analytical SEC. The functionality of purified CTD was probed by analyzing its binding interaction with purified CaM in the presence and absence of Ca^2+^ (EGTA-supplementation). The interaction had been described in detail with an X-ray structure of CaM with a bound fragment of CTD (residues 622–690)^21^. The presence of Ca^2+^ was required for a stable complex formation.

**Figure 2:**
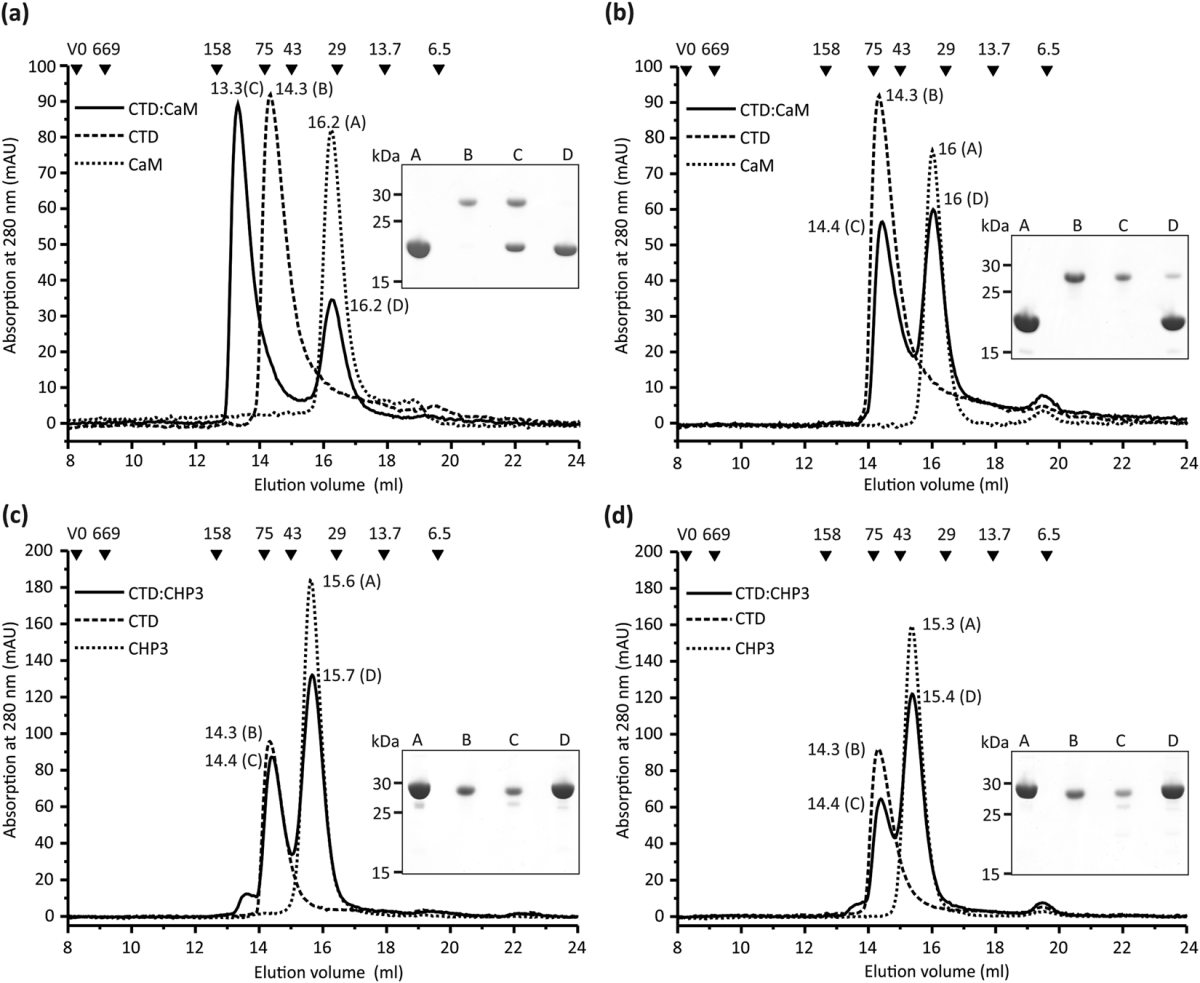
Interaction between CHP3, calmodulin and the CTD region of NHE1 probed *in vitro* by size exclusion chromatography. Experiment and analysis were performed as described in Fig. 1. Purified CTD and CaM were analyzed alone and as mixture with 2-fold molar ratio of CaM in the presence of 10 mM CaCl_2_ (**a**) and 10 mM EGTA (**b**). (**a**) The complex formation between CaM and CTD in the presence of Ca^2+^ is clearly demonstrated by a shift of the elution peak to a lower retention volume compared to the proteins alone. Both proteins are present in the respective elution fraction as shown by subsequent SDS-PAGE analysis. The peak D reflected residual CaM and indicates a stoichiometric interaction of CaM with CTD. (**b**) The binding interaction of CTD and CaM is Ca^2+^-dependent. Supplementation with EGTA abolished the shift of the elution peak to a lower retention volume thus demonstrating the lack of complex formation. (**c**) Analysis of the interaction between CTD and CHP3 in the presence of Ca^2+^ and (**d**) in the presence of 10 mM EGTA. Both chromatograms from the analysis of equimolar mixtures of CTD and CHP3 showed two elution peaks with largely unchanged retention volumes as compared to the analyses of the proteins alone. Thus, these two proteins do not form a stable complex, neither in the presence of Ca^2+^ or EGTA.

Highly pure and homogenous CaM preparations were obtained, as documented by a single narrow and symmetrical peak in the SEC elution profile and single bands in subsequent SDS-PAGE analysis of the respective elution fractions (Fig. 2a,b). The apparent molecular masses of 29 kDa and 30 kDa for Ca^2+^ (Fig. 2a) and Ca^2+^-depleted (Fig. 2b) samples are higher compared to 18.1 kDa theoretical molecular mass of His-tagged CaM. Larger SEC elution volumes were previously reported^24^ and a larger hydrodynamic radius agrees with the bi-lobed molecule shape of the molecule resolved in X-ray structures of CaM^25,26^. CaM and CTD were mixed in 2:1 molar ratio for SEC analysis as two CaM binding sites were reported for the CTD region^21,22^. In the presence of Ca^2+^, CaM binding was evident as the single elution peak of CTD (elution volume 14.3 ml, apparent molecular mass 28 kDa) was replaced with an elution peak of the CTD:CaM complex, which eluted at 13.3 ml corresponding to an apparent molecular mass of 109 kDa (Fig. 2a). Complex formation was proven by subsequent SDS-PAGE analysis of the eluted peak fractions, which showed the presence of both proteins. Notably, a second elution peak at 16.2 ml appeared which was assigned to CaM by SDS-PAGE analysis of the eluted peak fractions (Fig. 2a). Based on the respective peak areas, about half of the CaM remained unbound, indicating that a 1:1 CaM:CTD complex is resolved in SEC. This reflects most likely the interaction through the high affinity binding site (*K*_D_ = 20 nM, residues 637–656^21^). The low affinity site (*K*_D_ = 300 nM, residues 657–700) may not result in complex formation that is stable during SEC. The data are in line with the X-ray structure of the complex^21^.

To exclude non-specific binding interactions, the experiment was repeated in the presence of the highly selective Ca^2+^ chelator EGTA challenging the Ca^2+^ specificity of the binding (Fig. 2b). SEC analysis of CTD and CaM alone showed single peaks in the elution profiles also in EGTA supplemented buffer. The calcium-dependent binding of CaM supports the specific nature of the binding interaction with CTD.

Having confirmed the functionality of recombinant CTD, the interaction between CHP3 and CTD was investigated accordingly. CHP3 and CTD were mixed in 1:1 stoichiometric ratio and analyzed by SEC in the presence and absence of Ca^2+^ (Fig. 2c,d). In both conditions, single peaks in the elution profiles of CHP3 and CTD were resolved for proteins alone as well as for the mixture. In SDS-PAGE analysis of the eluted peak fractions, the individual proteins alone were resolved. Thus, it can be safely inferred that no complex formation occurred. In contrast to CaM, CHP3 does not bind to the CTD region of NHE1. This finding contrasts previous data obtained by Far-Western blot analysis that indicated interaction of CHP3 with a CTD peptide^17^, but is in line with the GST-NHE1-peptide pull-down experiments of CHP3 from reticulocytes lysates^13^.

### CHP3 binds to CBD with nanomolar binding affinity

**Figure 3:**
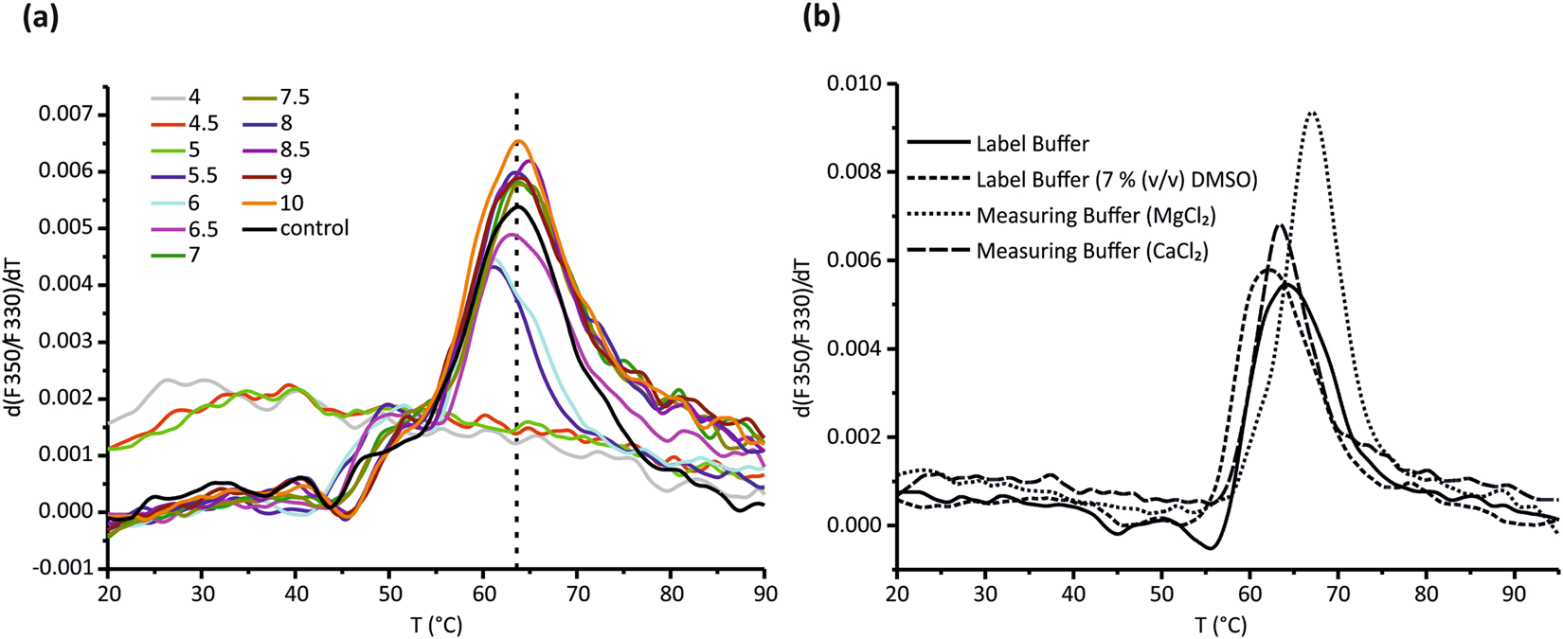
Optimization of room temperature stability of CHP3 for MST analysis. (**a**) Thermal stability of CHP3 in dependence of pH was analyzed by nanoDSF. CHP3 samples were diluted in succinic acid/sodium phosphate monobasic monohydrate/glycine buffers of defined pH (pH values are listed in the color code inset). A control buffer was used at physiological pH (20 mM Hepes pH = 7.2, 150 mM NaCl). Changes in fluorescence upon increasing temperature were monitored and the first derivative of the fluorescence ratio at 350 nm and 330 nm (F350/F330) is shown (one representative curve from three measurements). At and below pH 5, CHP3 melting curves indicated aggregation and lacked a defined melting point. Above, the melting points range from 61.1 to 64.3 °C. The melting point of 63.6 °C of the control buffer is indicated as dashed line. (**b**) Thermal stability of CHP3 was analyzed in MST labelling and measuring buffer probing the effect of supplementation with DMSO, Ca^2+^ and Mg^2+^. The following melting points were determined: labelling buffer, 63.9 °C; labelling buffer with 7% DMSO 61.7 °C; measuring buffer with MgCl_2_ 67.1 °C; measuring buffer with CaCl_2_ 63.4 °C.

Further, the binding interaction between CHP3 and CBD was quantitatively characterized making use of the available pure proteins. Buffer conditions were optimized to ensure protein stability during interaction analysis at room temperature. To screen for optimal pH conditions, a succinic acid/sodium phosphate monobasic monohydrate/glycine buffer system was chosen to isolate changes in pH from additional buffer effects^27^. Melting points were determined by nano-differential scanning fluorimetry (nanoDSF). His-tagged CHP3 which has a calculated isoelectric point of 5.0, aggregated at and below pH 5 (Fig. 3a). Above, the protein was stable in a wide pH range of pH 5.5 to pH 10 with melting points between 61 °C and 64 °C. The highest melting points were between pH 7 and pH 9. Thus, a physiological pH value of 7.2 could be a chosen for analysis.

The equilibrium dissociation constant (*K*_D_) of the interaction was determined by microscale thermophoresis (MST). The fluorescent label NT647 was attached to CHP3 enabling a direct control of CHP3 stability in solution. The standard labeling protocol had to be adjusted. CHP3 is prone to dimerization by disulfide bond formation^11^. Above pH 8, deprotonation may foster cysteine oxidation^28^. Thus, the pH of the labeling buffer was shifted to pH 7.6 and supplemented with tris(2-carboxyethyl)phosphine (TCEP) to prevent oxidation of CHP3. In addition, dimethyl sulfoxide (DMSO), which is typically used to dissolve the labeling dye, can also destabilize proteins and catalyze cysteine oxidation^29,30^. Indeed, addition of 7% of DMSO to the labelling buffer lowered the melting point of CHP3 by 2 °C (Fig. 3b). DMSO was thus omitted, the labeling dye directly dissolved into labeling buffer and the labelling carried out as described in materials and methods.

**Figure 4:**
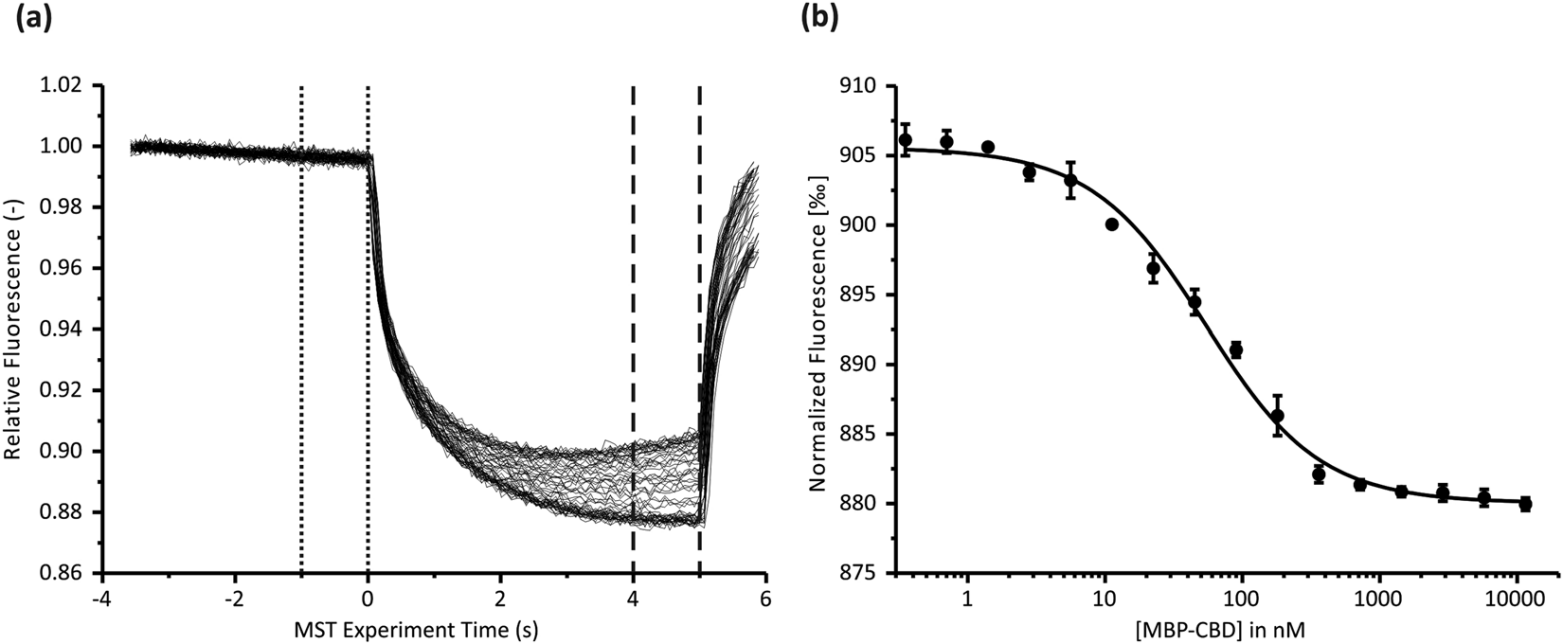
CHP3 binds with high affinity to the CBD region of NHE1 as probed *in vitro* by microscale thermophoresis. (**a**) Representative MST data for CHP3:MBP-CBD interaction showing superimposed time traces of five independent titration series for one biological replicate. The fluorescence was normalized to a starting value of 1. The laser was switched off after 5 s and the thermophoresis + T-jump signals were used for data analysis. Time spans for F_c_ and F_h_ are indicated with dotted and dashed lines, respectively. (**b**) MST binding curve derived from (**a**) as described in Materials and Methods. The line shows the best fit using the bimolecular binding model. The data for three biological replicates are summarized in Table 1. A mean *K*_D_ = 55.5 ± 6.8 nM (mean ± standard deviation (SD)) was determined.

The buffer composition was then optimized for MST measurements. CHP3 can bind Ca^2+^ and also Mg^2+^ ^11^. The effect of Mg^2+^ and Ca^2+^ on thermal stability of CHP3 was analyzed by nanoDSF. Supplementation of the buffer with Mg^2+^ increased the melting point of CHP3 by 4 °C, whereas Ca^2+^ addition had no effect on the melting point (Fig. 3b). Thus, MST measurements were performed in the presence of 10 mM MgCl_2_. Additional optimization steps included choice of laser power and type of capillaries and final conditions are described in the methods section. MST measurements were performed with three biological replicates, each of them measured with five technical replicates (Fig. 4 and Table 1). Positive thermophoresis was observed upon onset of the infrared laser and the measurement continued until the fluorescence reached a baseline. A representative MST measurement of one biological replicate is shown in Fig. 4. Thermophoresis data were collected for 5 s to avoid interference with convection. The mean *K*_D_ of the interaction of CHP3 with MBP-CBD was determined to be 56 ± 7 nM (±standard deviation (SD)).

**Table 1:** Quantitative analysis of binding interaction between CHP3 and CBD region of NHE1 by thermophoresis.

sample	*K*_D_ in nM	Bound (F_norm_ ‰)	Unbound (F_norm_ ‰)	Target conc. in nM	SD (*K*_D_) of five technical replicates per sample in nM	K_D_ confidence interval in nM
1	62.7	882.9	916.0	10	0.9	± 5.2
2	54.8	879.4	916.4	10	1.0	± 5.4
3	49.0	880.0	905.5	10	0.8	± 5.3
$\bar{{\rm{x}}}$ ± SD	55.5 ± 6.8					

$\bar{{\rm{x}}}$ ± SD = mean of three biological replicates ± standard deviation.

**Figure 5:**
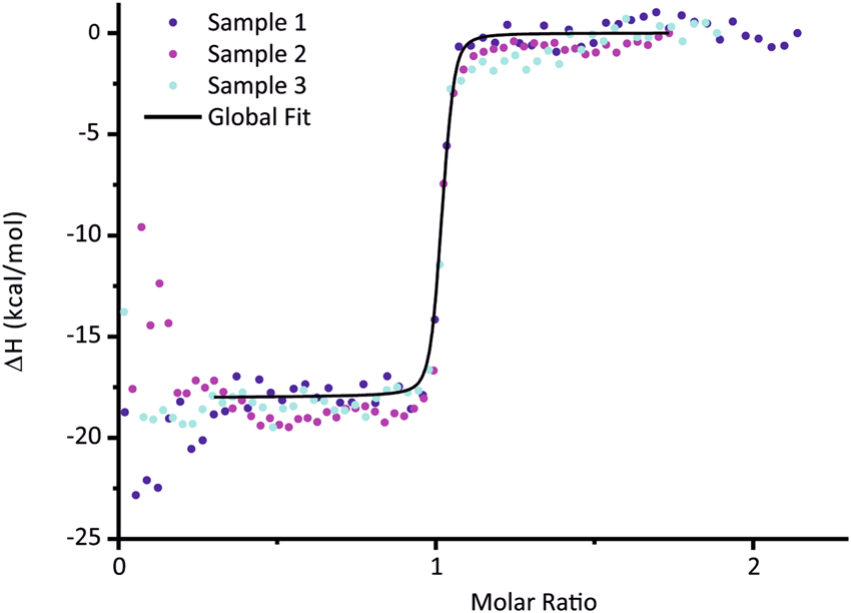
Analysis of the protein-protein interaction between CHP3 and MBP-CBD using isothermal calorimetry. Results of ITC experiments with biological triplicates, injecting CHP3 into MBP-CBD at 25 °C. The curves were subjected to a triple-shift correction (see ESI for details) assuming n = 1 and yielding *K*_D_ = 3.0 nM, 〈ΔH〉 = −75.3 kJ/mol, and individual active concentrations [CHP3] and [MBP-CBD] (see Supplementary Table S1). Data in the beginning of the experiments suffered from increased baseline noise and were excluded from the fit (see range of black curve of global fit).

The thermodynamic properties of the CHP3:MBP-CBD binding interaction were then analyzed by isothermal calorimetry (ITC). Assay conditions were kept highly similar to the MST assay, taking advantage of the assay optimization by MST that needed lower amount of protein as compared to the ITC measurements. A single difference in buffer composition was omission of the detergent Tween-20 in the ITC experiments to avoid foam formation during measurement. Figure 5 shows a compilation of three measurements performed with independent protein preparations and after correcting for concentration errors. Uncorrected data and the logics and procedure of the correction are detailed in the Supplementary Methods online. Briefly, typically the enthalpy change (ΔH) is fitted on the basis of the concentration of active CHP3 ([CHP3]) and stoichiometry (n), based on [CHP3] and [MBP-CBD]. We reversed this process based on the rationale that all samples should share the same ΔH and obey n = 1 for active CHP3 per active CBD. Subject to significant variations between the samples are not ΔH and n, but the active [CHP3] and [MBP-CBD] during the timespan of the ITC measurement. We have therefore employed a technique that assumes n = 1 and ΔH = constant and have quantified, instead, the average enthalpy, 〈ΔH〉, [CHP3], [MBP-CBD], and K_D_.

The *K*_D_ determined from a global fit of the three, concentration-corrected biological replicates was 3.0 nM with a 95% confidence interval of 2.5–3.7 nM (see support plane analysis in Supplementary Fig. S2), and 〈ΔH〉 = −75.3 kJ/mol. All results of individual and global fits are collected in Supplementary Table S1. The data showed that the interaction was exothermic with a strong enthalpic contribution. The *K*_D_ implies a standard Gibbs free energy change, ΔG°, of −48.6 kJ/mol and, along with 〈ΔH〉, an unfavorable, entropic contribution of −TΔS° = 26.7 kJ/mol.

## Discussion

The binding interaction of CHP3 with the regulatory domain of NHE1 was characterized in solution using highly purified protein preparations of CHP3 as well as of the CBD and CTD regions of NHE1. For CBD, the N-terminal fusion of a maltose-binding protein enabled yield and stability of the protein preparations, whereas hexa-histidine tags were sufficient for the others. SEC analysis showed binding of CHP3 to the proximal juxtamembrane region of NHE1’s regulatory domain comprising residues 503 to 545 (CBD), but not to its C-terminal part comprising residues 633–815 (CTD). The latter was functional in respect to binding of CaM in a Ca^2+^-specific manner. CHP3 formed a stoichiometric complex with the MBP-CBD fusion protein. The complex was stable in SEC analysis indicating a tight binding interaction. These data clearly support the view that CHP3 binds to the same region of NHE1 as CHP1 and CHP2. Structural characterization of the latter showed a 5-turn amphipathic helix composed of residues 518–537 of NHE1 docked into the hydrophobic pocket of CHP1^20^ and of CHP2^19^. In line with predominantly hydrophobic binding interactions, decreasing hydrophobicity for residues 526–531 by site-directed mutagenesis prevented formation of a stable complex between CBD variant (CBDcon) and CHP3.

Our data corroborate the results of pull-down assays, in which purified Glutathione S-transferase fusion proteins with NHE1 residues 505–571 interacted with ^35^S-CHP3 protein synthesized *in vitro* in reticulocyte lysates, but not with NHE1 residues 566-627^13^. The data are in contrast to previous findings, which reported binding of CHP3 to CTD (called His182 in that study) based on affinity blotting and fluorescence quenching^17^. In the former assay, CTD was separated by SDS-PAGE, transferred onto nitrocellulose and then positively decorated with CHP3. Decoration with CaM showed that the CaM binding site of CTD was functional in that assay. In the latter assay, CTD was labelled with a fluorescent probe that responds to the polarity of the environment and an addition of CHP3 quenched the fluorescence of labelled CTD. One should note that for CaM, two binding sites with *K*_D_ values of 20 nM and 350 nM were reported^22^. One cannot exclude that the interaction of CHP3 with CTD is so weak that it cannot be detected in size-exclusion chromatography or in pull-down experiments, though they are typically used to identify protein-protein interactions. The physiological relevance of the interaction of CHP3 with CBD was supported by co-immuno-precipitation and co-immunolocalization with *in vivo* experiments in Chinese hamster ovary A-1 cells. Simultaneous mutation of four hydrophobic residues within the discussed amphipathic helix of NHE1 blocked the interaction not only *in vitro* but also *in vivo* in intact cells^13^. In addition, the study showed that overexpression of CHP3 up-regulated the cell surface activity of NHE1 and this stimulation was not observed in the CHP3 binding-defective mutant. It is therefore of interest to characterize the physiological relevant binding interaction of CHP3 with the canonical CHP binding site of NHE1 in detail to elucidate the molecular basis of the modulation of NHE1 activity by CHP3.

As a first step towards this aim, the energetics of the binding interaction between CHP3 and CBD of NHE1 were characterized. Extensive assay optimization for the quantitative binding analysis made use of thermal profiling using nanoDSF and took advantage of the lower material consumption of the MST analyses as compared to ITC. For MST measurements, CHP3 was randomly labelled with a fluorescent probe. The deviations in respect to biological and technical replicates were low. A *K*_D_ of 56 nM at physiological pH of 7.2 was determined by MST. This is in line with the presence of stable complexes of CHP3 and MBP-CBD in SEC analysis. ITC analysis confirmed the strong interaction with a nanomolar affinity, a *K*_D_ of 3 nM was determined for the interaction. The addition of the detergent Tween-20 in the MST buffer, which was omitted in the ITC measurements, might be a reason for the observed difference. In addition, kinetic effects may have an impact on the determination of the equilibrium dissociation constant^31^. The high affinity of the interaction agrees with the co-localization of CHP3 and NHE1 shown *in vivo*^13^.

The thermodynamic parameters were measured with ITC and noteworthy, the binding interaction was strongly exothermic. This is on first sight surprising as one would assume a similar binding mode as in CHP1 and CHP2, in which the binding of the amphipathic helix of CBD is dominated by extensive hydrophobic interactions with a large hydrophobic cleft^19,20^. Hydrophobic interactions are almost exclusively of entropic nature at room temperature^32^. Yet, the peptide of the CBD region appears to be disordered as shown by circular dichroism and NMR analysis, and binding induced helix formation as shown for CHP1^20^. The thermodynamic parameters not only reflect the binding of the amphipathic helix to a hydrophobic pocket, but a strong enthalpic contribution is likely to derive from the transition of the disordered structure to the helix, for which values of −0.7 kcal/mol were described per residue folding into a helix^33^. The helix formation must thus be considered to dominate the overall, exothermic binding enthalpy. A comparison of thermodynamic parameters for the binding interaction with CHP1 and CHP2 would be interesting.

In conclusion, the study provides a detailed and quantitative characterization of the binding interaction of NHE1 and CHP3 in respect to binding site, energetics and thermodynamic parameters of the binding. The data aid the understanding of the molecular mechanisms that underlie the regulatory interactions with NHE1, processes that are essential for control of intracellular ion homeostasis and cell volume. Future studies have to address the effects of calcium ions and of CHP3 myristoylation on the interaction.

## Material and Methods

All column materials if not otherwise noted were obtained from GE Healthcare.

### Cloning and mutagenesis

The gene TESC (GC12M117038, Gene ID: 54997) encoding human CHP3 (UniProtKB Q96BS2) was cloned into the vector pBADM-11 (EMBL-AG) (pBADM11-*CHP3*), downstream of the sequence for N-terminal hexa-histidine tag and TEV cleavage site (Glu-Asn-Leu-Tyr-Phe-Gln\Ser) to yield the protein product Met-Lys-6xHis-Pro-Met-Ser-Asp-Tyr-Asp-Ile-Pro-2xThr-Glu-Asn-Leu-Tyr-Phe-Gln-Ser-Ala-Gly-Ser-Met-Lys-Gln-CHP3_HUMAN_2–214_. For production of human calmodulin (GC14P090396/Gene ID: 801), the expression plasmid pQE-T7_*CAM1* was commercially obtained (Qiagen). It encodes the final product Met-Lys-6xHis-Met-Lys-Gln-CALM1_HUMAN_2–149_. A synthetic sequence encoding residues 1–815 of SLC9A1 (GC01M027109/Gene ID: 6548) was used to amplify the corresponding sequences for NHE1-CBD (residues 503–545) and NHE1-CTD (residues 633–815) by PCR. The coding sequence for the CTD region was cloned via Gibson assembly^34^ into the vector pRSF13 (EMBL-AG) to generate a C-terminal hexa-histidine tag resulting in the protein product Met-NHE1_633–815_-6xHis (CTD) (pRSF13-*SLC9A1*^CTD^). The coding sequence for CBD was assembled with *mal*E (Gene ID: 948538), which encodes the *E. coli* maltose-binding protein (MBP; UniProtKB P0AEX9), and with sequences for TEV cleavage site and C-terminal octa-histidine tag in the vector pBADM-11 (pBADM11-*mal*E-*SLC9A1*^CBD^) to produce the protein product Met-MBP_27–393_-3xSer-10xAsn-Leu-Gly-Glu-Asn-Leu-Phe-Gln-2xGly-NHE1_503–545_-8xHis (MBP-CBD). The control construct was generated by site-directed mutagenesis to obtain MBP-CBDcon with residues Gln_526_-Gln-Asp-His-Gln-Gln_531_ substituted in the CBD region (pBADM11-*mal*E-*SLC9A1*^CBDcon^).

### Production and Purification of CHP3

Pre-cultures (200 ml 2XYT medium supplemented with 100 mg/l carbenicillin (2XYT^carb^)) were started with single colonies of pBADM11-*CHP3* transformed *E. coli* BL21 (DE3) cells, which had been grown overnight at 37 °C on LB-agar plates supplemented with 100 mg/l carbenicillin. Cultures were incubated overnight at 37 °C (220 rpm) and then added to 2 l 2XYT^carb^ medium to yield OD_600_ = 0.05. The main cultures were incubated at 37 °C (180 rpm) and protein production was induced at an OD_600_ = 0.5–0.7 with 0.02% L-Arabinose. After 6 h incubation at 30 °C, the cells were harvested by centrifugation and stored in aliquots at −80 °C.

25 g thawed cells were resuspended in 125 ml lysis buffer (20 mM HEPES, 500 mM NaCl, 20 mM imidazol, 5 mM DTT, 1 mM EDTA; pH = 7.2) supplemented with 1 mM Pefabloc (Carl Roth GmbH + Co. KG). Cells were lysed by passing the suspension three times through a cell disruptor (Constant Systems Ltd.) at 35 kPSi. Cell debris was removed by centrifugation (40 min at 35,000 g) and the supernatant was loaded onto a HisTrap HP 5 ml column pre-equilibrated with buffer A (20 mM HEPES, 500 mM NaCl, 20 mM imidazol, 5 mM DTT, 1 mM EDTA; pH = 7.2). Unbound proteins were removed with buffer A and the protein was eluted with a linear gradient of imidazole from 20 mM to 600 mM in buffer A over 10 column volumes. The eluted fractions were pooled, mixed with the same volume of SEC buffer (20 mM HEPES, 150 mM NaCl, 20 mM DTT, pH = 7.2) and concentrated using an Amicon Spin concentrator (10,000 MWCO) (Merck Millipore). The concentrated protein was loaded onto a HiLoad Superdex 75 16/60 column for the final purification by SEC. CHP3 and all other purified proteins of this study were stored in aliquots at −80 °C until use.

### Production and purification of MBP-CBD and MBP-CBDcon

Production and purification of MBP-CBD and MPB-CBDcon was performed essentially as described for CHP3 using the plasmids pBADM11-*mal*E-*SLC9A1*^CBD^ and pBADM11-*mal*E-*SLC9A1*^CBDcon^ with two modifications during SEC. The SEC buffer was supplemented with 1 mM TCEP (pH 7.2) and SEC was performed using a Hiload Superdex 200 16/60 column.

### Production and purification of CTD

Pre-cultures (200 ml 2XYT medium supplemented with 50 mg/l kanamycin (2XYT^kan^)) were started with single colonies of pRSF13-*SLC9A1*^CTD^, transformed *E. coli* BL21 (DE3) cells, which had been grown overnight at 37 °C on LB-agar plates supplemented with 50 mg/l kanamycin. Cultures were incubated overnight at 37 °C (220 rpm) and then added to 2 l 2XYT^kan^ medium to yield OD_600_ = 0.05. The main cultures were incubated at 37 °C (180 rpm) and protein production was induced at an OD_600_ = 0.5–0.7 with 1 mM IPTG. After 4 h incubation at 30 °C (180 rpm), the cells were harvested by centrifugation and aliquots stored at −80 °C. The purification was performed as described for MBP-CBD.

### Production and purification of CaM

The production of CaM using the plasmid pQE-T7_*CAM1* was performed as described for CTD with the modification, that incubation temperature was lowered to 30 °C after induction. The purification of CaM was carried out as described for CHP3, with the modifications that 1 mM CaCl_2_ was added to the SEC buffer and DTT was excluded.

### Analytical Size-Exclusion Chromatography

For the binding analysis between CHP3 and MBP-CBD as well as CHP3 and MBP-CBD^con^, all proteins were transferred into SEC elution buffer (20 mM HEPES, 150 mM NaCl, 1 mM TCEP; pH = 7.2) using a PD Minitrap G25 column. Protein samples were diluted to 20 µM each and 250 µl per protein was used to make an equimolar mixture, whereas 500 µl per protein was used for analysis of single proteins. 500 µl sample was injected to a 500 µl sample loop onto a Superdex 200 10/300 GL column and separated by SEC with a flow rate of 0.3 ml/min. 250 µL elution fractions were collected for each absorption maxima. Proteins were precipitated using 500 µl ice-cold acetone, sedimented by centrifugation (21,000 g for 30 min) and dried completely. For SDS-PAGE analysis, the samples were resuspended in 100 µl 1× -LDS loading buffer (NOVEX, life technologies) supplemented with 100 mM DTT and analyzed on a 12% Bis-Tris Gel (life technologies).

The binding analysis between CaM and CTD as well as CHP3 and CTD was carried out in the same analytical SEC buffer system like described above, but supplemented with either 10 mM CaCl_2_ or 10 mM EGTA. Sample preparation and analytical SEC was performed as described above. The stoichiometric ratio was adjusted for CaM:CTD analysis. 250 µl of 40 µM CaM was mixed with 250 µl of 20 µM CTD, to test both CaM binding sites of CTD. 500 µl of a 40 µM CaM solution was loaded for single CaM analyses. A Superdex 200 increase 10/300 GL column was used for enhanced resolution.

Both columns were calibrated using the high and low molecular weight gelfiltration calibration kit (GE Healthcare). Blue Dextran 2000 was used to determine V_0_ and Thyroglobulin (M_R_ = 669 kDa), Aldolase (M_R_ = 158 kDa), Conalbumin (M_R_ = 75 kDa), Ovalbumin (M_R_ = 43 kDa), Carbonic anhydrase (M_R_ = 29 kDa), Ribonuclease A (M_R_ = 13.7 kDa) and Aprotinin (M_R_ = 6.5 kDa) were used as protein standards. The apparent molecular mass was calculated following the manufacturer’s protocol.

### NanoDSF analysis

Buffer optimization for CHP3 was performed using nanoDSF. Buffer of purified CHP3 aliquots (100 µl was exchanged using PD SpinTrap G25 columns according to the manufacturer’s instructions. The effect of the MST labeling buffer was analyzed in both the presence and absence of 7% (v/v) DMSO as well as with either 10 mM MgCl_2_ or 10 mM CaCl_2_. After buffer exchange, the samples were diluted to a concentration of 3 mg/ml with the respective buffer. The nanoDSF measurement was performed using the Prometheus NT.48 device (NanoTemper) with an excitation intensity of 30%.

For analyzing the effect of pH, the buffer of 180 µl purified CHP3 was exchanged using Spin-Trap G25 columns to 150 mM NaCl and 20 mM HEPES at pH = 7.2. The protein sample was then diluted to 3 mg/ml with this buffer system. The experiment was done using conditions E1-E12 of the RUBIC buffer screen (Molecular Dimensions). For sample preparation, 8 µl of protein solution was mixed with 42 µl of each respective screening solution.

In both measurements, each sample was measured in triplicates using Prometheus NT.48 Series nanoDSF Grade High Sensitivity capillaries (Nanotemper) with an excitation intensity of 30%. The measurements were done over a range of 20 °C to 95 °C with a heating rate of 1 °C/min.

### Microscale Thermophoresis

Fluorescence labelling of CHP3 had to be optimized to avoid dimerization of CHP3, which has two cysteine residues which are prone to oxidize at pH > 8^28^. 100 µl of CHP3 were thawed and the buffer was exchanged into labeling buffer (50 mM HEPES, 150 mM NaCl, 5 mM TCEP, pH = 7.6) using column A of the labeling kit (NanoTemper). The concentration of CHP3 in the labeling buffer was determined spectroscopically (NanoDrop 2000 spectrophotometer, ThermoFisher Scientific) and subsequently diluted to 40 µM. Freshly-prepared solution of the fluorescent dye NT647 (Nanotemper) in labeling buffer (200 mg/ml) was mixed with the protein solution in a 1:1 volume ratio. The mixture was incubated for 30 min at room temperature. The buffer was exchanged to the measuring buffer (20 mM HEPES, 150 mM NaCl, 0.05% Tween-20, 10 mM MgCl_2_, 5 mM TCEP; pH = 7.2) using column B of the labeling kit in accordance with the manufacturer’s instructions. 180 µl of MBP-CBD were thawed, and buffer exchanged into measuring buffer using a PD SpinTrap G-25 column.

Protein concentration as well as protein to dye ratio for MBP-CBD and CHP3 were determined using a UV-Vis spectrophotometer (Cary 4000, Agilent) at 280 nm and 650 nm, respectively. The extinction coefficients for the proteins were calculated using the ProtParam tool (ExPASy): CHP3, ε_280_ = 14,440 M^−1^ cm^−1^; MBP-CBD, ε_280_ = 69,330 M^−1^ cm^−1^. For the calculation of CHP3 to dye ratio, ε_650_ = 250,000 M^−1^ cm^−1^ was used for NT647 with a correction factor of 0.028 to compensate for the dye absorption at 280 nm according to manufacturer’s protocol. The measured labeling efficiency was determined to be about 30%.

The microscale thermophoresis (MST) based protein-protein interaction analysis between CHP3 and MBP-CBD was performed with the Monolith NT.115Pico device (NanoTemper). CHP3^NT647^ was diluted with measuring buffer to 20 nM concentration of CHP3. A serial dilution of MBP-CBD was prepared starting with 10 µl of MBP-CBD after buffer exchange and 10 µl of measuring buffer, and repeating this 1:1 dilution over 14 additional dilution steps. For 16 capillaries, 10 µl CHP3 (20 nM) was mixed with 10 µl of MBP-CBD samples each, covering the concentration prior to dilution and all dilution steps. To allow for binding equilibrium, mixed samples were incubated for 30 min at 4 °C followed by 10 min in a metal block at room temperature. The samples were then loaded into capillaries (Monolith NT.115 MST premium coated). Excitation was set to 10% and MST-Power to 40%. The initial state was recorded for 3 s, thermophoresis for 5 s and the back diffusion for 1 s. The assay was done with three independently produced and purified biological replicates per protein and five independent titration and measurement series per biological replicate (technical replicates). The measurements were done at 22 °C.

The measured data were analyzed with the MO. Affinity Analysis Software from the manufacturer (NanoTemper). Normalized fluorescence was calculated as *F*_norm_ = *F*_h_/*F*_c_ × 1,000. The technical replicates were averaged and fitted using the bimolecular binding model^35^ with fixed target protein concentration. The data were processed graphically using Origin Pro 2017 (OriginLab Corporation).

### Isothermal Calorimetry

For isothermal calorimetry (ITC) analysis, 250 µl aliquots of CHP3 and MBP-CBD were prepared by spin filtration (Amicon Ultra-4 with 10000 MWCO, Merck Millipore) to concentrations between 400–500 µM. The concentrated protein samples were then dialyzed (10 kDa, Mini Dialysis Cassette, Thermo Fisher) twice in 1 l of filtered ITC buffer (20 mM HEPES, 150 mM NaCl, 10 mM MgCl_2_, 5 mM TCEP; final pH = 7.2) for 2 hours at 4 °C. Protein concentration was measured spectroscopically (Nanodrop2000 UV-vis spectrophotometer). CHP3 and MBP-CBD were diluted to 200 µM and 20 µM, respectively, using the buffer of the second dialysis cycle.

The analysis was performed using the PEAQ-ITC device (Malvern). The reservoir was filled with 300 µl of 20 µM MBP-CBD, 70 µl of 200 µM CHP3 was loaded into the syringe. Titrations were performed with 57 injections per run at 25 °C for three biological replicates. Protein concentrations of the final samples were determined spectroscopically in triplicates, diluting 10 μl of protein sample with 90 μl of buffer solution (UV-vis spectrophotometer Cary 4000, Agilent). The data were analyzed using the Software from Malvern for PEAQ-ITC.

## Electronic supplementary material

Supplementary Information
