# Supplementary material for: Calcineurin B homologous protein 3 binds with high affinity to the CHP binding domain of the human sodium/proton exchanger NHE1
Source: Sci Rep. 2018 Oct 4;8:14837. doi: 10.1038/s41598-018-33096-5 (PMC6172220; doi:10.1038/s41598-018-33096-5)
Supplement: Supplementary file 1 — Supplementary Information [file 41598_2018_33096_MOESM1_ESM.pdf]

Supplementary Information

Calcineurin B homologous protein 3 binds with high affinity to the CHP binding domain of the human sodium/proton exchanger NHE1

Simon Fuchs[1,2], Sierra C. Hansen[1], Marie Markones[3,4], Evgeny Mymrikov[1,4], Heiko Heerklotz[3,4,5], Carola Hunte[1,4]*

[1] Institute for Biochemistry and Molecular Biology, ZBMZ, Faculty of Medicine, University of Freiburg, D-79104 Freiburg, Germany; [2] Faculty of Biology, University of Freiburg, D-79104 Freiburg, Germany; [3] Department of Pharmaceutical Technology and Biopharmacy, University of Freiburg, D-79104 Freiburg, Germany; [4] BIOSS Centre for Biological Signalling Studies, University of Freiburg, D-79104 Freiburg, Germany; [5]Leslie Dan Faculty of Pharmacy, University of Toronto, Canada

[*] Correspondence: carola.hunte@biochemie.uni-freiburg.de

Supplementary Methods

Global fit of ITC data obtained with biological replicates: the triple-shift approach

Affinity, binding enthalpy, and stoichiometry of binding could be determined from a proper ITC curve with high precision, if the exact concentrations of active, binding competent proteins (here: [CHP3], [MBP-CBD]) were known and if the resolution suffices to resolve the sigmoidal shape (with several points in the steep part). The latter problem can be solved by smaller injections (sacrificing heat signal) and by considering more than one measurement globally. Then, the by far dominant source of error arises from the determination of protein concentrations by UVvis spectrophotometry and potential fractions of inactive protein.

A robust and scientifically rigorous solution to this problem is the global fit of measurements from biological triplicates. They should share the same ideal fit parameters (n, K_D, ΔH) but are subject to different errors of [CHP3] and [MBP-CBD]. For our experiments - with CHP3 in the syringe and MBP-CBD in the cell - the error of active [CHP3] will lead to an error in ΔH and n. An error in [CBD] will only affect n. This suggests the following “triple-shift” procedure:

1) All data sets are fitted freely, yielding individual values of K_D, ΔH_i^{raw}, n, and the offset or blank, Q_0.
2) The three values of ΔH_i^{raw} are averaged to yield $\langle \Delta H \rangle$ and the individual concentration settings for the replicates, $[CHP3]_I^{raw}$ (i=1, 2, 3) are corrected as:

   $$[CHP3]_i^{corr} = [CHP3]_i^{raw} \cdot \frac{\Delta H_i^{raw}}{\langle \Delta H \rangle} \qquad (0.1)$$

   which renders the individual concentrations used in the fit, $[CHP3]_I^{corr}$ mutually consistent (but a general, systematic error might remain).
3) Now, the individual fits of the curves are repeated using the individual, corrected $[CHP3]_I^{corr}$ values and fixing ΔH to $\langle \Delta H \rangle$. This brings the height of the sigmoidal curves to a common value of $\langle \Delta H \rangle$ (shift 1). Different fit results are obtained for n (now termed n_i^{raw}) and K_D.

Deviations of n_i^{raw} from the nominal value of 1 are now used to correct the concentrations of the cell content for each experiment, $[MBD\text{-}CBD]_i$:

4) $$[\text{MBP-CBD}]_i^{corr} = [\text{MBP-CBD}]_i^{raw} \cdot n_i^{raw} \quad (0.2)$$

5) Repeating the fits now with set values of $[CHP3]_i^{corr}$, $[MBP\text{-}CBD]_i^{corr}$, and $\langle \Delta H \rangle$ should yield n=1, i.e. it matches the points of inflection (shift 2), and provides an improved value of K_D.
6) The corrected data sets can now be fitted globally (effectively increasing the number of points at the sigmoidal "step" and improving statistics in general. Using the MALVERN ITC software, we did not perform a global fit but a manual support plane analysis, which is simple since only K_D needs to be varied at this stage[1]. For all 3 curves, we determined χ^2 as a function of pre-set K_D over a range around the value of the individual fits. Then, we plotted the sum of these χ^2 / χ^2_{min} values as a function of K_D, fitted empirically by a fourth degree polynomial, yielding a minimum at 3.0 nM. The 95% confidence interval is then indicated by the range where $\chi^2/\chi^2(\text{min}) < 1.01005$ (see Supplementary Fig. S2).
7) In order to graphically overlay the curves as done in Fig. 5 (main text), the individual offsets (Q^0 of final fit) can be subtracted (shift 3).

Table S1: Results of individual ITC data as well as a global fit following the triple-shift procedure as outlined in the text above.

Raw data					
i	1	2	3	average	STD
[CHP3] (µM)	200	200	200	200	0
[MBP-CBD] (µM)	20	20	20	20	0
n	0.97	1.2	1.1	1.09	0.11
K_D (nM)	3.0	2.4	4.5	3.3	1.1
ΔH (kcal/mol)	-18.6	-15.0	-20.3	-18.0	2.7
Offset	-2.4	-8.8	-10.7	-7.3	4.3
Corrected					
[CHP3] (µM)	207	167	226	200	30
[MBP-CBD] (µM)	20.1	20.0	24.9	21.7	2.8
n	1	1	1	1.0	0.0
K_D (nM)	3.1	2.1	5.1	3.4	1.5
Offset	-2.4	-10.5	-9.4	-7.4	4.4
⟨ΔH⟩ (kcal/mol)	-18.0	-18.0	-18.0	-18.0	0.0
global					
				95% confidence interval	
K_D (nM)	3.0			2.6 – 3.5	

This procedure suggests that it would be advantageous to fill the component into the syringe the active concentration of which is more precisely known. Other constraints such as available material or solubility may, however, require a different approach.

Alternative approaches following the same logics are available, for instance the global fit analysis in sedphat[2].

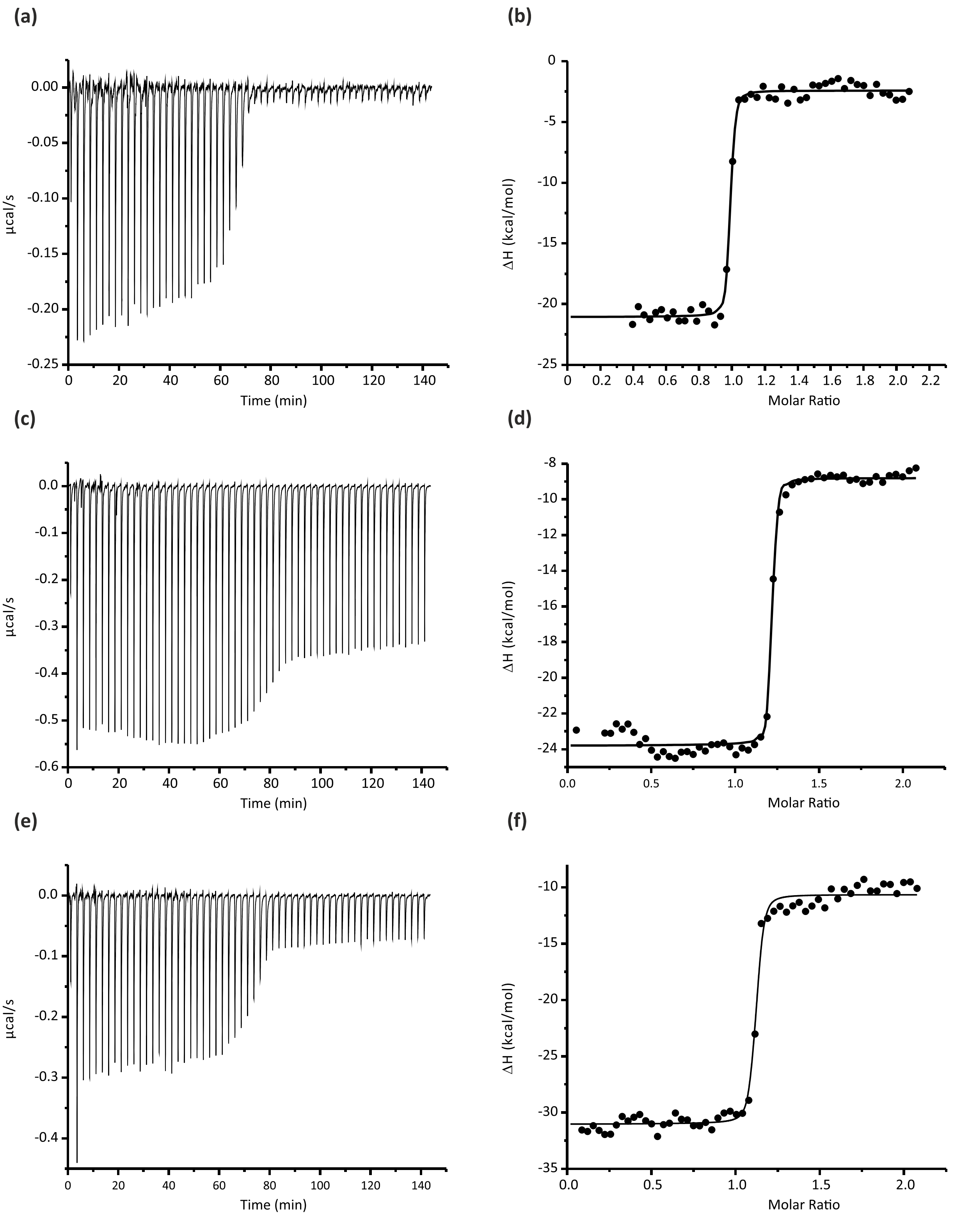

Figure S1. Thermograms with arbitrary baseline subtracted (left) and integrated ITC curves (right) for biological triplicates (top, middle, bottom) of CHP3:MBP-CBD binding experiments at 25°C. These curves were fitted individually (see parameters in Supplementary Table S1) as well as subjected to the triple-shift procedure to derive figure 5 (main text) and a global fit of K_D.

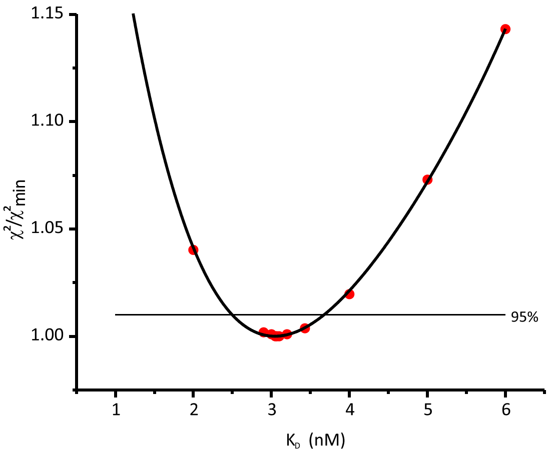

Figure S2. Support plane analysis for the global fit of K_D to the three data sets shown in Fig. 5, assuming n=1 and corrected concentrations of CHP3 and MBP-CBD as listed in Supplementary Table S1. Red dots show the ratio of the sum of χ^2 of biological triplicates at a certain K_D divided by the minimal χ^2 at K_D = 3.0 nM. Black line shows empirical fit of forth degree polynomial, thin black line indicates the 95% confidence interval.

Reference

1 Kemmer, G. & Keller, S. Nonlinear least-squares data fitting in Excel spreadsheets. *Nat Protoc* **5**, 267-281, doi:10.1038/nprot.2009.182 (2010).

2 http://www.analyticalultracentrifugation.com/sedphat/isothermal_titration_calorimetry.htm on Sep 01/2018
